# KRAS G12C and KRAS G12D respond to lipid metabolism in an allele-specific manner

**DOI:** 10.1016/j.jlr.2026.101079

**Published:** 2026-06-16

**Authors:** Neha Arora, Hong Liang, Walaa Kattan, Wantong Yao, Haoqiang Ying, Junchen Liu, Yong Zhou

**Affiliations:** 1 Department of Diagnostic and Biomedical Sciences, School of Dentistry, University of Texas Health Science Center, Houston, Texas, USA; 2 Department of Integrative Biology and Pharmacology, McGovern Medical School, University of Texas Health Science Center, Houston, Texas, USA; 3 Division of Pathology-Lab Medicine Div, Department of Translational Molecular Pathology, The University of Texas MD Anderson Cancer Center, Houston, Texas, USA; 4 Division of Basic Science Research, Department of Molecular and Cellular Oncology, The University of Texas MD Anderson Cancer Center, Houston, Texas, USA; 5 Program of Molecular and Translational Biology, Graduate School of Biological Sciences, M. D. Anderson Cancer Center and University of Texas Health Science Center, Houston, Texas, USA

**Keywords:** KRAS, nanoclustering, lysophosphatidylcholine acyltransferase 1, phosphatidylserine, acyl chains, electron microscopy, cancer biology, phospholipids

## Abstract

*KRAS* mutated at hotspots G12, G13, and Q61 possess profound allele-specific oncogenesis. Signaling of KRAS mutants is mostly compartmentalized to the proteolipid nanoclusters on the plasma membrane (PM), illustrating critical roles of spatiotemporal organization in KRAS cancer signaling. The activated GTP-bound KRAS molecules, including the wild type and mutants, have been traditionally thought to favor similar lipids. We recently reported distinct lipid sensing capabilities of different KRAS mutants, especially with KRAS^G12D^ favoring unsaturated lipids and KRAS^G12C^ gaining additional enrichment of saturated lipids. As such, KRAS mutants may respond to lipid acyl chain remodeling in an allele-specific manner. Lysophosphatidylcholine acyltransferase 1 (LPCAT1) facilitates the incorporation of saturated fatty acid chains to phospholipids. Here, we found that cells stably expressing *LPCAT1* contain higher levels of saturated lipids and lower levels of unsaturated PS species. Electron microscopy–spatial analysis revealed that *LPCAT1* expression disrupts the nanoclustering of KRAS^G12D^ on the PM, without affecting that of KRAS^G12C^ and HRAS^G12V^. Elevation of *LPCAT1* expression suppresses signaling, proliferation, and colony formation of KRAS^G12D^-expressing human pancreatic cancer cells, while promoting those of KRAS^G12C^-expressing cells. Knocking out *LPCAT1* depletes saturated lipids and reduces colony formation of KRAS^G12C^ cells. We further found that changing *LPCAT1* expression specifically targets *KRAS* mutant-expressing cells, without affecting cells expressing WT *KRAS*. Mouse embryonic fibroblasts transformed with *KRAS*^*G12C*^ also contain more saturated lipids than *KRAS*^*G12D*^ MEFs. Thus, activities of KRAS mutants depends on lipid acyl chain remodeling in an allele-specific manner.

KRAS4B (or KRAS) small GTPase is a molecular switch that toggles between the inactive GDP- and active GTP-bound states (1, 2, 3, 4). KRAS activates a wide variety of signaling cascades, especially MAPKs, and regulates cell survival, growth, division, proliferation, and migration (1, 2, 3, 4). Constitutively active mutants of KRAS at hotspot residues G12, G13, and Q61 in its enzymatic globular G-domain (amino acids 1–166) are major drivers of cancer, contributing to 98% of pancreatic, 45% of colorectal and 31% of lung tumors (1, 2, 3, 4). KRAS mutants display remarkable allele- and tissue-specific oncogenic activities. Mutations G12D and G12V are dominant mutations in >70% of pancreatic and >50% of colorectal cancers (5). G12C is a minor mutation in pancreatic and colorectal cancers (<4%) and a dominant driver in ∼30% of lung tumors (5). In vitro assays further illustrate that KRAS mutants possess allele-specific effector binding and GTP hydrolysis (6). It is not clear how KRAS mutants require different cancer cell environments to sustain their oncogenic activities.

A hallmark of KRAS oncogenesis is the compartmentalization of its signaling to nano-domains enriched with distinct lipids (7, 8), signifying the pathological importance of its spatial regulation. The C-terminal lipid-anchored membrane-anchoring domain of KRAS has been largely attributed as the main lipid-sensing feature (2). However, all its enzymatic and pathogenic activities are determined by the G-domain, which remains largely cytosolic. Interestingly, the KRAS G-domain undergoes allosteric reorientation when anchored to the membranes, sampling two main orientation states (OSs), each of which presents a distinct membrane interface (9, 10, 11, 12, 13, 14, 15, 16). We recently reported that mutating key basic residues at these membrane interfaces of KRAS differentially alters the preference of acyl chain structures of phosphatidylserine (PS) lipids (17). We further illustrated that KRAS oncogenic mutants, which possess distinct allosteric OS equilibria (18, 19, 20), enrich distinct plasma membrane (PM) lipids (17). KRAS^G12D^, KRAS^G12V^, and KRAS^Q61H^ selectively favor mixed-chain PS, while KRAS^G12C^ and KRAS^G13D^ gain additional association with saturated PS, cholesterol and/or phosphoinositol 4,5-bisphosphate (PIP_2_) (17). The distinct lipids enriched in these nanoclusters are functionally important because effectors of KRAS possess their own lipid-recognizing motifs and require synergistic binding of both KRAS and select lipids for efficient PM association and signal propagation (1, 2, 21, 22). Thus, activities of KRAS mutants may respond to perturbations of lipid metabolism in distinct manners.

Because of the distinct preferences of KRAS^G12C^ and KRAS^G12D^ for saturated versus unsaturated lipids described above, we compared how remodeling homeostasis of saturated/unsaturated lipids differentially impacts activities of KRAS^G12C^ versus KRAS^G12D^. Lysophosphatidylcholine acyltransferases (LPCAT1) preferentially catalyzes the generation of saturated lipids, such as dipalmitoylphosphatidylcholine (23, 24). We now show that cells stably expressing *LPCAT1* contain higher levels of saturated phosphatidylcholine (PC) and phosphatidylethanolamine (PE), and lower levels of major mixed-chain PS species. *LPCAT1* expression more effectively disrupts PM association, signaling and oncogenic activities of KRAS^G12D^, while not affecting or promoting those of KRAS^G12C^. KRAS^G12C^-expressing cells with *LPCAT1* knocked out contain lower levels of saturated lipids and their ability to form colonies is reduced. Taken together, oncogenic activities of KRAS mutants depends on lipid metabolism in an allele-specific manner.

## Materials and methods

### Electron microscopy (EM)-univariate nanoclustering

Apical or basolateral PM of baby hamster kidney (BHK) or human pancreatic tumor MiaPaCa-2 cells expressing GFP-KRAS^G12V^ or GFP-HRAS^G12V^ was attached to electron microscopy (EM) grids. The intact native PM sheets were then fixed with 4% paraformaldehyde/0.1% gluaraldehyde, tagged with anti-GFP antibody conjugated with 4.5 nm gold nanoparticles, and negative stained with 0.3% uranyl acetate, and embedded in methyl cellulose. Transmission EM was used to image intact PM sheets at 1,000,00× magnification. ImageJ was used to assign the x/y coordinates of each gold particle within a select 1 μm^2^ PM area. A univariate Ripley’s K-function tested a null hypothesis that the gold nanoparticles distribute in a random pattern:

(A) $$K\left(r\right)=An^{-2}\sum_{i\neq j}w_{ij}1\left(\left\|x_{i}-x_{j}\right\|\leq r\right)$$

(B) $$L\left(r\right)-r=\sqrt{\frac{K\left(r\right)}{\pi }}-r$$

In Equation A, *K(r*) denotes the univariate distribution for gold nanoparticles with a total number of *n* in a PM area of *A*; *r* signifies the distance between gold particles with an increment of 1 nm from 1 to 240 nm; || ^.^ || denotes Euclidean distance that describes an indicator of 1(^.^) = 1 if ||*x*_*i*_-*x*_*j*_|| ≤ r and 1(^.^) = 0 if ||*x*_*i*_-*x*_*j*_|| > r. *w*_*ij*_^-1^ is used to correct edge effects by describing the fraction of the circumference of a circle with the center defined as *x*_*i*_ and radius ||*x*_*i*_-*x*_*j*_||. In Equation B, *L*(*r*) – *r* denotes the linear transformation of *K*(*r*) in Eq. A, which is achieved by normalizing *K*(*r*) against the 99% confidence interval (99% C.I.) calculated via Monte Carlo simulations. *L*(*r*) - *r* values between a 99% confidence interval (99% CI) of 1 and -1 indicate random pattern of spatial distribution. *L*(*r*) - *r* values above 99% CI indicate statistically meaningful clustering, with larger *L*(*r*) - *r* values describing more extensive clustering. *L*(*r*) - *r* values below −1 indicate declustering. The peak values of *L*(*r*) - *r* curves, termed as *L*_*max*_, are used as a summary statistic to signify the extent of nanoclustering. For each condition, at least 15 PM sheets from individual cells were imaged and analyzed. Statistical significance was evaluated via comparing our calculated point patterns against 1000 bootstrap samples in non-parametric bootstrap tests (25, 26).

### Cell culturing and generation of stable lines

Human and murine pancreatic tumor cell lines, including MOH, PANC1, BxPC3, and iKRAS cells, were maintained in DMEM medium containing 10% FBS). PDAC cell line MiaPaCa-2 was maintained in DMEM medium containing 10% FBS and 2.5% horse serum. To generate stable cell lines, the pEF6 vector plasmid without/with the cDNA of human LPCAT1 was used to transfect the tumor cells. For each line, 1 μg of plasmid was added to 7 μl of lipofectamine for the transfection. Following 5-h incubation with the plasmids, cells were washed and changed to DMEM medium containing 10% FBS and 3 μg/ml puromycin antibiotic. Cells were grown in the presence of antibiotics for a week before serial dilution and seeding in 96-well plates with a concentration of < 1 cell per well. Cell colonies were then harvested for Western blotting to verify the expression of LPCAT1.

### Western blotting

Whole-cell lysates of MOH, PANC1 and BxPC3 cells were collected. Following electrophoresis in SDS PAGE gels and transfer, membranes were incubated with primary antibodies against the phosphorylated ERK and Akt, total ERK and Akt, LPCAT1, as well as loading control of actin, overnight. After secondary antibody incubation, membranes were imaged using ECL solution. Data are shown as mean ± SEM. ImageJ software analysis was used to evaluate expression intensity and identify fold change.

### Proliferation

CyQUANT cell proliferation assay was used to measure number of live cells in microplates. Appropriate number of PDAC cells, such as MOH (1000 cells/well), MiaPaCa-2 (2000 cells/well) and BxPC3 (3000 cells/well), were seeded in 96-well plates. After 96 h, cells were washed and stained with CyQUANT® GR dye. Following lysis, fluorescence of dye bound to intact nucleic acids was measured using a Tecan plate reader. For each condition, 3 independent experiments were performed. Student’s *t* test was used to evaluate the statistical significance.

### Colony formation

All human and murine pancreatic cancer cell lines stably expressing V2 or LPCAT1 were seeded in 6-well plates. Specifically, iKRAS (400 cells/well), MOH (250 cells/well) cells were grown for 7 days. MiaPaCa-2 (100 cells/well) were grown for 10 days and BxPC3 (1000 cells/well) were grown for 14 days. Cells were washed twice with PBS, followed with fixation with 4% paraformaldehyde for 15 min. Cell staining was performed with 0.01% crystal violet for 15 min. Colony images were captured using PerkinElmer X3 multiplate reader. Colony count was performed using ImageJ. For each condition, 3 independent experiments were conducted. Student’s *t* test was used to evaluate the statistical significance.

### Lipidomics

Human PDAC MiaPaCa-2 and BHK cells were grown to confluency and whole-cell lysates were collected at a concentration of 1.8 × 10^6^ cells/ml in a volume of 500 μl, which was frozen at −80°C. Samples were shipped to Lipotype GmbH (Dresden, Germany) on dry ice for lipidomics analysis. Over 100 lipid types and ∼4200 individual lipid species were standardized against internal standards. Three individual experiments were analyzed for each condition. Data were averaged and shown as mean ± SEM, and Student’s *t* test was used to evaluate the statistical significance.

## Results

### LPCAT1 elevates saturated phospholipids and depletes unsaturated phospholipids

**Fig. 1:**
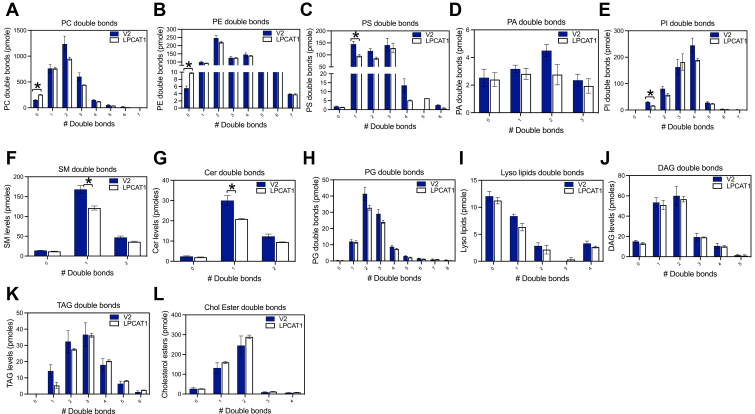
LPCAT1 expression elevates the saturated lipids in mammalian cells. Whole-cell lysates of baby hamster kidney (BHK) cells stably expressing *V2* empty vector or *LPCAT1* were collected for shotgun lipidomics. Levels of lipid species with different numbers of double bonds (in p moles) for phosphatidylcholine (PC, A), phosphatidylethanolamine (PE, B), phosphatidylserine (PS, C), phosphatidic acid (PA, D), phosphoinositols (PI, E), sphingomyelins (SM, F), ceramides (Cer, G), phosphatidylglycerol (PG, H), lysophospholipids (I), diacylglycerols (DAG, J), triacylglycerols (TAG, K), and cholesterol esters (Chol Esters, L) are shown as mean ± SEM from 3 independent trials. Student’s *t* test was used to evaluate the statistical significance, with ∗ indicating *P* < 0.05. LPCAT1, lysophosphatidylcholine acyltransferase 1.

**Fig. 2:**
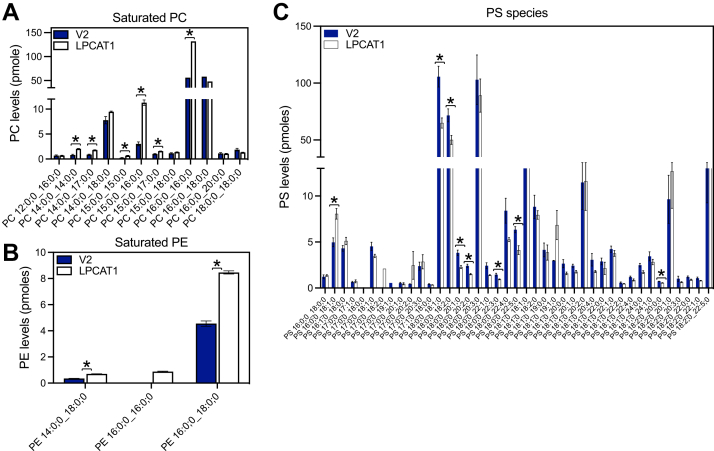
LPCAT1 alters homeostasis of main phospholipid types. Stable expression of *V2* or *LPCAT1* elevated the levels of multiple saturated PC (A) and PE (B) species. C: Effects of V2 or Lpcat1 expression on the levels of all the PS species are shown. Data are shown as mean ± SEM from 3 independent trials. Student’s *t* test was used to evaluate the statistical significance, with ∗ indicating *P* < 0.05. LPCAT1, lysophosphatidylcholine acyltransferase 1; PC, phosphatidylcholine; PE, phosphatidylethanolamine.

We generated BHK cells and human PDAC MiaPaCa-2 cells stably expressing empty vector *V2* or *LPCAT1*. Changes in LPCAT1 expression were validated in Western blotting (supplemental Fig. S1A). Subsequent shotgun lipidomics (Fig. 1A-L) illustrated that BHK cells expressing *LPCAT1* contained significantly higher levels of saturated PC and PE species (Fig. 1A, B), especially di14:0 PC, di16:0 PC, 14:0/18:0 PE and 16:0/18:0 PE (Fig. 2A, B), consistent with previous studies (23, 27). Furthermore, cells stably expressing *LPCAT1* contained reduced levels of mixed-chain phospholipids with monounsaturated *sn-2* acyl chains, especially PS, phosphoinositols, SM and ceramides (Fig. 1). We then focused on PS species since KRAS spatial distribution is particularly sensitive to PS lipids. As shown in Fig. 2C, various major mixed-chain PS species, such as 18:0/18:1 PS, 18:0/18:2 PS, 18:0/20:1 PS, 18:0/20:2 PS, 18:0/22:3 PS, and 18:0/22:5 PS, were significantly lower in BHK cells expressing *LPCAT1* than V2 vector control.

LPCAT1 stable expression in MiaPaCa-2 cells (expression validated in Supplemental Fig. S1B) modulated lipidome in a similar manner as BHK cells (Supplemental Fig. S2). Especially, MiaPaCa-2 cells expressing *LPCAT1* contained higher levels of saturated PC and PE, especially 14:0/16:0 PC, di16:0 PC, 14:0/16:0 PE and 14:0/18:0 PE, when compared to vector control (Supplemental Fig. S3A, B). In MiaPaCa-2 cells with *LPCAT1* stable expression, levels of major mixed-chain PS species with monounsaturated and polyunsaturated *sn-2* chains, such as 18:0/18:1 PS, 18:0/18:2 PS and 18:0/18:3 PS, were also lower (Supplemental Fig. S3C). Taken together, LPCAT1 expression in mammalian cells significantly modulates homeostasis of saturated/unsaturated phospholipids.

### LPCAT1 differentially impacts the PM association of KRAS mutants

**Fig. 3:**
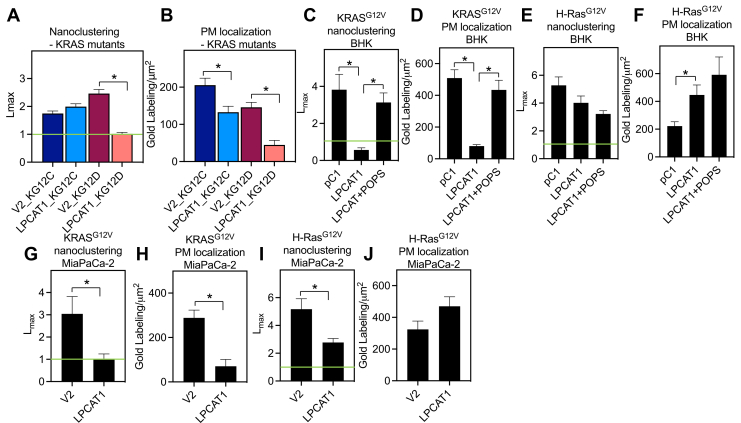
LPCAT1 preferentially disrupts the signaling nanoclustering of KRAS^G12D^ than KRAS^G12C^. Spatial distribution of KRAS mutants was quantified via electron microscopy (EM)-spatial analysis. Intact apical PM sheets of BHK cells stably expressing *V2* or *LPCAT1* transiently expressing GFP-KRAS^G12C^ or GFP-KRAS^G12D^ were attached to copper EM grids. GFP anchored to the PM inner leaflet was immunolabeled with anti-GFP antibody conjugated to 4.5 nm gold nanoparticles. Distribution of the gold-labeled GFP-KRAS^G12C^ and GFP-KRAS^G12D^ within a selected 1 μm^2^ PM area was calculated using the Ripley’s K-function analysis. A nanoclustering curve was plotted as the extent of nanoclustering, *L*(*r*) – *r*, versus length scale, *r* in nanometers. The peak value of the curve, termed as *L*_*max*_, was used as a summary statistic to indicate nanoclustering (A). The *L*(*r*) – *r* of 1 is the 99% confidence interval (99% CI, green line), the values above which indicate statistically meaningful clustering. Number of gold particles within the 1 μm^2^ PM area was counted to indicate PM localization (B). C, D, E, and F: BHK cells stably expressing *V2* or *LPCAT1* transiently expressing GFP-KRAS^G12V^ or GFP-HRAS^G12V^ were supplemented without/with 10 μM exogenous mixed-chain 16:0/18:1 PS (POPS) for 1 h before EM analysis. G, H, I, and J: MiaPaCa-2 cells stably expressing *V2* or *LPCAT1* transiently expressing GFP-KRAS^G12V^ or GFP-HRAS^G12V^ were also subjected to EM analysis. The summary statistic for nanoclustering *L*_*max*_ (A, C, E, G, and I) and gold labeling density (B, D, F, H, and J) are shown as mean ± SEM. For the nanoclustering data, the statistical significance was evaluated via the non-parametric bootstrap tests. For the gold labeling data, the statistical significance was quantified using the one-way ANOVA. ∗ indicates *P* < 0.05. BHK, baby hamster kidney; LPCAT1, lysophosphatidylcholine acyltransferase 1.

We recently reported allele-specific lipid sensing of KRAS oncogenic mutants, with KRAS^G12D^ and KRAS^G12V^ preferring mixed-chain PS and KRAS^G12C^ gaining association with saturated PS (17). To test how spatial distribution of KRAS mutants might differentially respond to lipid acyl chain remodeling, we compared effects of *LPCAT1* expression on the signaling nanoclusters of GFP-KRAS^G12C^ and GFP-KRAS^G12D^, using EM-univariate nanoclustering analysis. Briefly, BHK cells stably expressing V2 or LPCAT1 were ectopically transfected with a GFP-KRAS mutant. The apical PM sheets of these cells were attached to copper EM grids. GFP-KRAS mutants anchored to the PM inner leaflet were immunolabeled with anti-GFP antibody conjugated to 4.5 nm gold nanoparticles. Gold distribution was imaged via transmission EM at 1,000,00x magnification (sample EM images shown in Supplemental Fig. S4A–C). Spatial distribution of gold particles within a 1 μm^2^ PM area was quantified using the Ripley’s K-function analysis. The extent of nanoclustering, *L*(*r*) – *r*, was plotted against distance *r* in nanometers (Supplemental Fig. S4D, the gold particles in the same EM images were color-coded to indicate population distribution in Supplemental Fig. S4*E*–*G*). The peak *L*(*r*) – *r* value, or *L*_*max*_, was used as a statistical summary for the nanoclustering. *L*(*r*) – *r* values above the 99% confidence interval (99% CI) of 1 indicate the statistically meaningful nanoclustering, with larger *L*_*max*_ values corresponding to more extensive nanoclustering. The number of gold particles within the same1 μm^2^ PM area estimates the PM localization of GFP-KRAS. In Fig. 3A, *L*_*max*_ of GFP-KRAS^G12C^ was not affected by LPCAT1 expression. *L*_*max*_ of GFP-KRAS^G12D^ was markedly lower in BHK cells expressing *LPCAT1* than *V2* vector control, suggesting that the nanoclustering of KRAS^G12D^ is more sensitive to perturbation of *LPCAT1* expression. In Figure 3B, gold labeling of GFP-KRAS^G12C^ decreased by ∼ 35% in the *LPCAT1*-expressing cells when compared to V2 control, while gold labeling of GFP-KRAS^G12D^ decreased by ∼69% by *LPCAT1* expression. Taken together, the PM association of KRAS^G12D^ is more sensitive to *LPCAT1* perturbation than that of KRAS^G12C^.

We previously showed that KRAS^G12V^ and KRAS^G12D^ favor similar mixed-chain PS, while HRAS^G12V^ and KRAS^G12C^ partially lose selectivity for PS acyl chains (17). We next compared effects of LPCAT1 on the PM association of GFP-KRAS^G12V^ and GFP-HRAS^G12V^ in BHK cells. As expected, *LPCAT1* expression significantly disrupted the nanoclustering and PM localization of GFP-KRAS^G12V^, which was effectively restored by acute addback of mixed-chain 16:0/18:1 PS (POPS, Fig. 3C, D). LPCAT1 did not impact the nanoclustering of GFP-HRAS^G12V^, but increased the PM localization of GFP-HRAS^G12V^ (Fig. 3E, F). Acute addback of POPS did not alter the PM association of GFP-HRAS^G12V^ in BHK cells. In MiaPaCa-2 cells, *LPCAT1* stable expression disrupted the nanoclustering and PM localization of GFP-KRAS^G12V^ (Fig. 3G, H), consistent with its effects on KRAS^G12V^ in BHK cells. Interestingly, *LPCAT1* expression partially disrupted the nanoclustering of GFP-HRAS^G12V^ without affecting its PM localization (Fig. 3I, J). Taken together, the LPCAT1-induced lipid acyl chain remodeling differentially impacts the PM association of KRAS mutants in distinct manners.

### LPCAT1 impacts MAPK signaling and oncogenic activities of KRAS-dependent tumor cells in an allele-specific manner

**Fig. 4:**
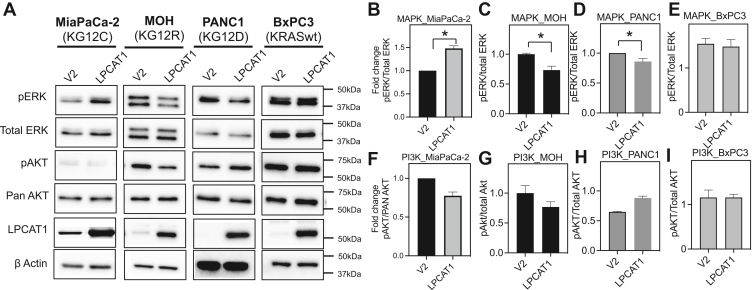
LPCAT1 differentially impacts the signaling and oncogenic activities of KRAS mutants in an allele-specific manner. (A) Whole-cell lysates of human pancreatic tumor lines, including MiaPaCa-2 (KRAS^G12C^), MOH (KRAS^G12R^), PANC1 (KRAS^G12D^) and BxPC3 (KRAS WT) stably expressing V2 or LPCAT1, were collected for Western blotting. Antibodies against the phosphorylated ERK (pERK), total ERK, pAkt, total Akt and LPCAT1 were used to blot for targeted proteins. Sample blots for a single trial are shown. Quantifications of pERK/total ERK for MiaPaCa-2 (B), MOH (C), PANC1 (D) and BxPC3 (E), as well as pAkt/total Akt for MiaPaCa-2 (F), MOH (G), PANC1 (H) and BxPC3 (I), are shown as mean ± SEM from 3 independent experiments. Statistical significance was evaluated using Student’s *t* test, with ∗ indicating *P* < 0.05. LPCAT1, lysophosphatidylcholine acyltransferase 1.

We next compared effects of LPCAT1 on MAPK and PI3K signal output in the KRAS-dependent human PDAC cell lines, including MiaPaCa-2 (KRAS^G12C^), MOH (KRAS^G12R^) and PANC1 (KRAS^G12D^) cells, and the WT KRAS-expressing BxPC3 cells. *LPCAT1* stable expression resulted in a ∼ 50% increase in the phosphorylated ERK (pERK/total ERK) in MiaPaCa-2 cells, significantly decreased pERK/total ERK levels in MOH and PANC1 cells, while having minimal effect on MAPK signaling in BxPC3 cells (Fig. 4A–E, complete blots shown in Supplemental Fig. S5). The PI3K signaling (pAkt/total Akt), which is less preferentially regulated by KRAS, was unaffected by *LPCAT1* expression (Fig. 4A, F–I).

**Fig. 5:**
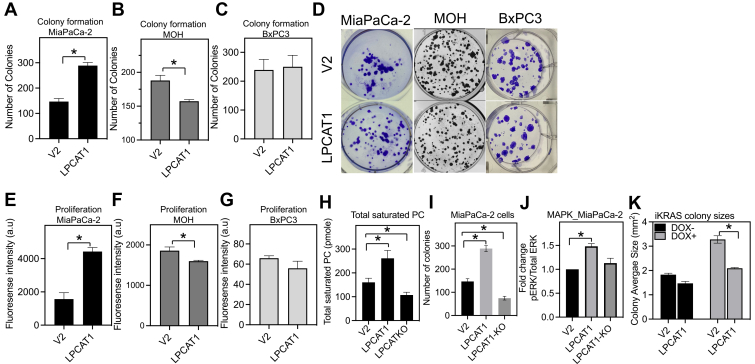
LPCAT1 differentially impacts oncogenic activities of tumor cells driven by different KRAS mutants. The KRAS-dependent and KRAS-independent human PDAC cells stably expressing *V2* or *LPCAT1* were seeded in 6-well plates. Colonies were counted after 96 h of growth. The number of colonies for MiaPaCa-2 (A), MOH (B), and BxPC3 (C) are shown as mean ± SEM from 3 independent trials. D: Sample images of MiaPaCa-2, MOH, and BxPC3 colonies are shown. To evaluate proliferation, MiaPaCa-2 (E), MOH (F), and BxPC3 (G) cells stably expressing *V2* or *LPCAT1* were seeded in 96-well plates. After 96 h of growth, CyQUANT cell proliferation assay was used to measure proliferation. To compare *LPCAT1* stable expression and knockout, MiaPaCa-2 cells transfected with vector control, *LPCAT1* or sgRNA of *LPCAT1* were subjected to lipidomics to compare levels of saturated lipids (H), MAPK signal output (pERK/total ERK) (I), and number of colonies (J). (K) An inducible KRAS^G12D^-driven PDAC iKRAS mouse line was used to evaluate specificity of *LPCAT1* expression. Murine pancreatic adenocarcinoma iKRAS cells (isolated from conditional KRAS^G12D^-expressing mice, p48Cre Kras^G12D L/+^p53^L/+^) were maintained in doxycycline (DOX+) to induce expression of KRAS^G12D^, or withdrawn from DOX for 48 h (DOX-) for KRAS independent condition. The iKRAS (DOX+/−) cells stably expressing vector control (*V2*) or *LPCAT1* were seeded in 6-well plates. After 96 h, sizes of the colonies were measured. Average colony size was initially measured in pixels using ImageJ, which was then converted to mm^2^. All colony formation, proliferation, signaling, and lipidomics data are shown as mean ± SEM from 3 independent experiments. Student’s *t* test was used to evaluate the statistical significance with ∗ indicating *P* < 0.05. LPCAT1, lysophosphatidylcholine acyltransferase 1.

We then compared effects of LPCAT1 on oncogenic activities of MiaPaCa-2, MOH and BxPC3 cells. When compared with the vector control, *LPCAT1* stable expression increased the number of colonies of MiaPaCa-2 cells, significantly reduced colony formation of MOH cells, while having no effect on BxPC3 cells (Fig. 5A–D). Similarly, *LPCAT1* stable expression increased proliferation of MiaPaCa-2 cells, decreased proliferation of MOH cells, while having no effect on BxPC3 cells (Fig. 5E–G). We next knocked out *LPCAT1* in MiaPaCa-2 cells (validated using Western blotting shown in supplemental Fig. S1, C, D). As expected, cells with *LPCAT1* stable expression contained higher levels of saturated PC, while cells with *LPCAT1*KO contained lower levels of saturated PC (Fig. 5H). Number of colonies for MiaPaCa-2 cells with *LPCAT1*KO was reduced when compared with the vector control, while colony formation of MiaPaCa-2 cells stably expressing *LPCAT1* was enhanced as shown above (Fig. 5I). Interestingly, *LPCAT1*KO did not affect MAPK signaling of MiaPaCa-2 cells (Fig. 5J). To further evaluate the specificity of LPCAT1 in impacting KRAS mutants, we stably expressed *V2* vector or *LPCAT1* in mouse PDAC iKRAS cells with inducible expression of KRAS^G12D^. The iKRAS tumor cells were isolated from genetically modified mice with conditional KRAS^G12D^ transgene expression (p48Cre Kras^G12D L/+^ p53^L/+^) (28). DOX treatment (DOX+) effectively induces expression of KRAS^G12D^, while DOX withdrawal (DOX-) for 24 h extincted KRAS^G12D^ expression (28). In Fig. 5K, LPCAT1 stable expression significantly decreased sizes of colonies of iKRAS lines induced to express KRAS^G12D^, while having minimal effects on iKRAS cells without KRAS mutant expression. Taken together, LPCAT1 differentially impacts signaling and oncogenic activities of KRAS-dependent tumor lines in an allele-specific manner, consistent with its allele-specific effects on the spatiotemporal organization of KRAS mutants.

### KRAS^G12C^- and KRAS^G12D^-expressing cells possess distinct lipidomes

**Fig. 6:**
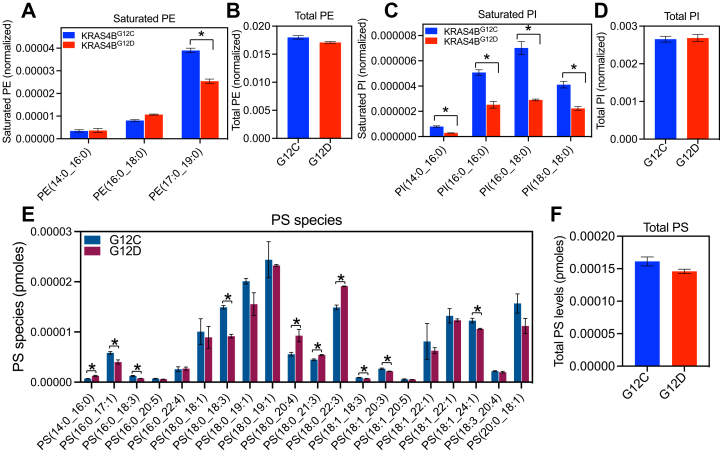
KRAS^G12C^-expressing cells contain higher levels if saturated lipids. Whole-cell lysates of RASless MEFs driven by KRAS^G12C^ or KRAS^G12D^ were collected for lipidomics. Normalized levels of saturated PE (A), total PE (B), saturated PI (C), and total PI (D) are shown. Individual species of PS lipids (E) and total PS (F) are also shown. All data are shown as mean ± SEM from 3 independent experiments. Student’s *t* test was used to evaluate the statistical significance with ∗ indicating *P* < 0.05. PE, phosphatidylethanolamine; PI, phosphatidyinositol; PS, phosphatidylserine.

Cancer cells have been shown to modify their lipidomes to sustain KRAS oncogenic activities (29, 30). To evaluate how cells expressing different KRAS mutants modulated their lipidomes, we compared lipidomes of RASless mouse embryonic fibroblasts (RASless MEFs) expressing KRAS^G12C^ or KRAS^G12D^. The RASless MEFs are isolated from mice with null NRAS and HRAS alleles and a floxed KRAS locus, then infected with Cre Recombinase Adenovirus carrying genes of specific KRAS mutants (31). With all endogenous RAS genes deleted and distinct RAS mutants expressed, RASless MEF lines are powerful tools for comparing pathophysiological activities of specific RAS mutants (31). Whole-cell lysates of RASless MEF lines expressing KRAS^G12C^ or KRAS^G12D^ were collected for lipidomics analysis. In Figure 6A–D, RASless MEF cells expressing KRAS^G12C^ contained higher levels of saturated PE and PI species than KRAS^G12D^-expressing RASless MEF cells, without affecting total PC and PE levels. For PS species, KRAS^G12C^-MEF cells contained distinct profiles of PS species than KRAS^G12D^-MEF cells (Fig. 6E), while sharing similar total PS levels (Fig. 6F). Specifically, KRAS^G12D^ cells contained higher levels of mixed-chain PS species with saturated *sn-1* chain and polyunsaturated *sn-2* chains, such as 18:0/20:4 PS, 18:0/21:3 PS and 18:0/22:3 PS than KRAS^G12C^ cells. On the other hand, KRAS^G12C^ cells contained more mixed populations of different types of PS species including dually unsaturated PS species (18:1/18:3 PS, 18:1/20:3 PS and 18:1/24:1 PS), in addition to mixed-chain PS species with a saturated *sn-1* chain (Fig. 6E). This is consistent with our previous findings that KRAS^G12D^ more selectively associates with mixed-chain PS with a saturated *sn-1* chain, while KRAS^G12C^ partially loses acyl chain selectivity (17). Taken together, the KRAS^G12D^-expressing cells more selectively enrich the mixed-chain PS species, while KRAS^G12C^-expressing cells partially lose such selectivity.

## Discussion

Here, we show that the LPCAT1-altered homeostasis of saturated/unsaturated lipids modulates oncogenic activities of KRAS mutants in distinct manners (summarized in Table 1). Lipid acyl chain structures, such as chain length and degrees of unsaturation, play critical roles in establishing local membrane core properties and lateral spatial segregation of lipids into functional domains. The most well-established lipid domains are lipid rafts, with saturated lipids, cholesterol and sphingolipids as main constituents and characterized by tighter packing and slower diffusion. On the other hand, unsaturated lipids enrich the more loosely packed and more mobile non-raft domains (17, 32). The distinct mesoscopic environments within these nano-domains facilitate the formation of various signaling scaffolds. KRAS has been traditionally considered as a non-raft-preferring protein (26, 33). On supported bilayers, the WT KRAS preferentially intercalates into the disordered domains enriched with unsaturated lipids, in the absence of saturated lipids and cholesterol (34). On the native cell PM, the WT, G12D and G12V mutants of KRAS with unsaturated lipids and segregate from saturated lipids and cholesterol (17, 25, 26, 33, 35, 36). The nanoclusters of these KRAS proteins are insensitive to cholesterol depletion (17, 33, 36). Incorporation of KRAS WT into lipid rafts in the cell PM, by replacing its C-terminal lipid anchor with that of HRAS (a raft-preferring protein), abolishes activation of its effector CRAF and MAPK signaling (37). Interesting, we recently reported that, unlike previously thought, some KRAS mutants, such as KRAS^G12C^ and KRAS^G13D^, prefer to associate with saturated lipids and cholesterol, and their PM association is disrupted by cholesterol depletion (17). We now show that elevating saturated lipids and reduction of unsaturated lipids by LPCAT1 expression promote signaling and function of KRAS^G12C^, while reducing those of KRAS^G12D^. These findings suggest that KRAS mutants possess distinct lipid sensing capability and display allele-specific dependency on lipid metabolism.

**Table 1:** A summary of KRAS allele-specific lipid sensing and allele-specific responses to LPCAT1 expression

Lipid types and parameters	KRAS^G12C^	KRAS^G12D^	KRAS^G12R^	KRAS WT
Lipid preference				
Saturated lipids	Yes^a^	No^a^	N/A	N/A
Cholesterol	Yes^a^	No^a^	N/A	N/A^b^
PIP_2_	Yes^a^	No^a^	N/A	N/A^b^
LPCAT1				
Nanoclustering	No effect	Down	N/A	N/A
MAPK signaling	Up	Down	Down	No effect
PI3K signaling	No effect	No effect	No effect	No effect
Proliferation	Up	Down	Down	No effect
Colony formation	Up	Down	Down	No effect

Lipid sensing and responses to LPCAT1 expression of KRAS proteins, including mutants G12C, G12D, G12R, and WT, are compared in mammalian cells.

a Data obtained from Arora et al.

b Data obtained from Zhou et al.

It is not clear how the largely cytosolic G-domains of KRAS mutants can sense lipid acyl chains embedded within the bilayer core. A possible mechanism may be coordinated conformational shifts of their G-domains and lipid anchoring domains. Abankwa *et al.* (38, 39) first proposed a balance model describing how the HRAS G-domain samples distinct conformational states when anchored to the membranes. HRAS bound to GDP anchors to the membranes mainly using its hypervariable region (HVR), with its G-domain extended away from the membranes. This conformational coordination allows the C-terminal palmitoyl chains to fully extent into the membranes and pack favorably with the tightly packed saturated lipids. When GTP-bound, the HRAS G-domain rotates toward the membrane, with charged residues on helix α4 forming hydrogen bonds with lipid headgroups in the membranes. The extensive contact of the G-domain with the membranes in turn pulls its HVR palmitoyl chains partially out of the membranes, causing the palmitoyl chains to become disordered and pack more favorably with the more fluid regions of the membranes enriched with unsaturated lipids. Recent studies demonstrated that the KRAS G-domain undergoes allosteric reorientation between an open OS1 and an occluded OS2, defined by how the effector-binding pocket is exposed or obstructed by the membranes (10, 11, 14, 40, 41). Oncogenic mutations of KRAS, even at the same site, shift OS1/OS2 equilibria (10, 42, 43, 44). For example, the negatively charged aspartate in G12D introduces additional electrostatic interactions that stabilizes OS1 state (45, 46, 47). The hydrophobic side chain of valine residue in G12V creates additional steric hindrance, whereas the cysteine substitution in G12C introduces a thiol group that creates additional structural distortion in the surrounding regions of the G-domain (45, 46, 47). OS1 and OS2 present distinct membrane interfaces. We showed OS1 and OS2 facilitate lipid sensing in distinct manners (17). Interestingly, the polybasic lipid-anchoring domain of KRAS is predicted to adopt distinct conformations in OS1 versus OS2 (16). Thus, the polybasic domain and the G-domain of KRAS may coordinate in a similar fashion as the G-domain and lipid anchor of HRAS in the balance model. It is, thus, possible that the coordinated conformational shifts of the G-domains and lipid anchors of G12C and G12D mutants facilitate the observed allele-specific lipid sensing.

A key purpose of select lipid enrichment is formation of the signaling nanoclusters to facilitate efficient effector recruitment and signal transduction. Major KRAS effectors, especially BRAF and CRAF, contain their own PS-binding motifs and localize to the PM in a PS-dependent manner (22, 48). PS depletion mislocalizes BRAF and CRAF from the PM with distinct efficiencies (22). We further showed that CRAF recruitment by KRAS^G12V^ occurs only in the presence of mixed-chain PS but not saturated PS (26). Furthermore, a polybasic domain mutation K177Q flips the lipid preference of KRAS from PS to PIP_2_, which in turn causes KRAS^K177Q^ to more effectively activate PI3K/Akt than MAPK (Zhou *et al. Cell* 2017). Our current findings are also consistent with this concept. The LPCAT1-induced elevation of saturated lipids (PC and PE) and depletion of mixed-chain lipids (PC and PS) disrupt signaling and activity of the KRAS^G12D^-cells, while promoting those of KRAS^G12C^-cells. These observations are consistent with the lipid-dependent effector recruitment. As such, the ability of KRAS to recruit different effectors can also depend on distinct lipids enriched within their nanoclusters. For example, PIP_2_ enriched by KRAS^G12C^ (17) may facilitate better recruitment of the PIP_2_-binding PI3K and the activation of Akt signaling, when compared with the PS-enriched KRAS^G12D^ and KRAS^G12V^. This potential mechanism requires further investigation.

We, here, observed that MEF cells expressing KRAS^G12C^ contain higher levels of saturated lipids than those expressing KRAS^G12D^. The KRAS^G12D^-expressing cells also contain higher levels of mixed-chain PS species. This is consistent with the allele-specific lipid enrichment of these KRAS mutants. Similar phenomena have been observed in previous studies. Specifically, Caco2 cells expressing KRAS^G12V^ contain higher levels of mixed-chain PS than Caco2 cells expressing HRAS^G12V^ (29). Acutely, it is possible that KRAS mutants sequester distinct lipid species into their signaling nanoclusters, preventing these lipids from being degraded, thus elevating their levels in cells. Interestingly, previous studies showed that, in cells transformed by KRAS mutants, expression of components in lipid biosynthesis and transport is modulated accordingly. For instance, oxysterol-related binding proteins ORP5 and ORP8 localize to the PM/ER contact sites and facilitate exchange of phosphoinositol 4-phosphate in the PM with PS lipids in the ER. ORP5 and ORP8 mRNA expression is higher in patient PDAC tumor samples containing KRAS mutants, when compared with patient samples containing WT KRAS (49). Similarly, expression of Eighty-Five Requiring, homolog A (EFR3A), a scaffold protein for the synthesis of phosphoinositol 4-phosphate , is higher in PDAC cells transformed by KRAS mutants (50). Expression of elongation of very-long-chain fatty acids family member 6 (ELOVL6, a fatty acid elongase that facilitates the synthesis of mixed-chain PS species) correlates with expression of KRAS mutants in tumor cells (30). Taken together, cells transformed by KRAS oncogenic mutants fundamentally reprogram their lipid metabolism and transport to sustain spatial regulation and cancer signaling of these KRAS mutants.

It is possible that cancer cells expressing different KRAS mutants may express LPCAT1 differently to sustain their allele-specific activities. We analyzed two patient databases in the Genomic Data Commons: The Cancer Genomic Atlas (TCGA)-pancreatic adenocarcinoma (PAAD), and TCGA-lung adenocarcinoma (LUAD). Supplemental Fig. S6A shows that *LPCAT1* mRNA levels were significantly higher in PAAD patients with WT *KRAS* than those with mutant *KRAS*, suggesting that *LPCAT1* expression negatively correlates with KRAS oncogenesis in PAAD. *LPCAT1* expression did not correlate with *KRAS* mutation status in LUAD patients (Supplemental Fig. S6B). Concordantly, *LPCAT1* and *KRAS* expression levels displayed a negative correlation in PAAD (Supplemental Fig. S6C) but no correlation in LUAD (Supplemental Fig. S6D). KRAS^G12D^ and KRAS^G12V^ are dominant mutant alleles (46% and 31%, respectively) in human PAAD, while KRAS^G12C^ occurs less frequently (3%). In non-small cell LUAD, on the other hand, KRAS^G12C^ (37%), KRAS^G12D^ (19%) and KRAS^G12V^ (22%) all occur at relatively similar rates. Thus, the negative correlation between LPCAT1 and KRAS mutant expression in PAAD and lack of correlation in LUAD are consistent with this view. Interestingly, LPCAT1 protein levels in cancer patients showed a different pattern. In Supplemental Fig. S6E, primary tumors of PAAD contained higher LPCAT1 than normal pancreatic tissue. Similar higher LPCAT1 proteins were also found in tumor samples of LUAD patients (Supplemental Fig. S6F). Transcript levels do not always correlate with protein expression. Importantly, the mRNA and proteomic analyses address distinct biological comparisons. TCGA RNA-seq analyses compare LPCAT expression among PDAC tumors stratified by KRAS mutation status and reveal lower *LPCAT1* mRNA expression in KRAS mutant tumors relative to the rare KRAS WT PDAC cases. In contrast, CPTAC proteomic data accessed through UALCAN compare tumor versus normal tissue. An additional factor likely contributing to these differences in that the proteomics and transcriptomics datasets are derived from distinct patient cohorts with differences in sample composition and tumor purity. This may explain the differences in these data analyses. Furthermore, our experimental findings regarding an allele-specific response suggest that LPCAT1 may have context-dependent effects on cell growth, which could further explain why a direct correlation is not observed across these distinct cohorts.

*LPCAT1* expression has been shown to promote oncogenic activities of epidermal growth factor receptor (EGFR) by elevating levels of saturated lipids (24). EGFR prefers to localize to lipid rafts, proteolipid clusters enriching saturated lipids, cholesterol, PIP_2_ and sphingolipids (32). This reflects the complex biological and pathological roles of LPCAT1 and intricate selectivity of lipid acyl chain remodeling. EGFR dimerization/oligomerization occurs in lipid rafts, which in turn promotes autophosphorylation and signaling (24, 51). KRAS^G12C^ distributes to similar raft domains since the KRAS mutant favors to associate with saturated lipids, cholesterol and PIP_2_ (17), consistent with its signaling and oncogenic activities being promoted upon *LPCAT1* expression. On the other hand, KRAS^G12D^ and KRAS^G12V^ favor mixed-chain PS species (17). Consistently, we now show that the LPCAT1 expression attenuates the nanoclustering and signaling of KRAS^G12D^ and KRAS^G12V^. Thus, the opposing effects of LPCAT1 on the cholesterol-dependent EGFR and KRAS^G12C^ versus the cholesterol-poor KRAS^G12D^ and KRAS^G12V^ suggest potential selectivity, with which targeting lipid metabolism can achieve.

The lipid-mediated intracellular transport and lateral nanoclustering of membrane components are typically interconnected properties. While often coordinated in their responses to membrane perturbations, the two parameters occasionally decouple. For instance, our current study shows that *LPCAT1* expression reduces abundance of KRAS^G12C^ on the PM, as a component of intracellular transport, without affecting its nanoclustering. We previously showed that acute addback of dual-unsaturated PS species promotes the PM localization, but not the nanoclustering, of KRAS^G12V^ (26). Furthermore, PM depolarization enhances the nanoclustering of the WT and G12V mutant of KRAS without affecting its PM abundance (25). Cholesterol depletion mislocalizes KRAS^G13D^ from the PM, without affecting its nanoclustering (17). Transport and lateral distribution of lipids can also decouple. Elevation of membrane curvature elevates the PM abundance of dual-saturated and dual-unsaturated PS lipids, without affecting their lateral nanoclustering (35). We previously formally tested whether the nanoclustering of KRAS depends on its abundance without direct external perturbation (35). We altered abundance of KRAS molecules on the PM of BHK cells by changing KRAS expression levels. Despite its immunogold labeling levels on the PM increases over three orders of magnitude (∼40–∼1500 gold-labeled KRAS molecules/μm^2^ of PM area), KRAS nanoclustering remains completely independent of its expression levels (35). Although thermodynamic models dictate that population distribution of molecules depends on their abundance in the system, it is unclear how the two properties mechanistically coordinate and decouple in native cell membranes. A plausible explanation is that biological membranes feature relatively static lipid domains, which play a direct role in structuring and maintaining the spatial arrangement of membrane elements (52, 53, 54). We previously proposed that actin and caveolae control distinct immobile reservoirs of PS lipids in the native cell PM (52). PS lipids embedded within these tightly packed and immobile reservoirs are less accessible to some proteins. By releasing PS lipids into the mobile pools or trapping PS lipids within, these actin- and caveolae-dependent reservoirs actively maintain availability of lipids for biological activity, such as sustaining clustered fractions of KRAS on the cell PM (52). In support of this view, we previously showed that disassembly of caveolae, by knocking down caveolin 1, promotes the nanoclustering of PS lipids without affecting levels of PS lipids in the PM (55). Disruption of actin polymerization by Latrunculin treatment also increases the mobile fraction of PS lipids in the PM (52). Taken together, native cell membranes may possess various immobile lipid reservoirs that actively coordinate coupling between spatiotemporal organization and abundance of membrane constituents. This mechanism requires further comprehensive investigation.

## Conclusion

KRAS oncogenic mutants display allele-specific lipid sensing. We, here, show that perturbing biosynthesis of saturated lipids by genetically modifying LPCAT1 expression differentially impacts the nanoclustering, signaling, and oncogenic activities of KRAS mutants. Our findings suggest that the KRAS G-domain dynamics contribute to spatial regulation of its signaling and activities. Future studies may focus on characterizing how networks of lipid metabolism, such as complex interplay between different LPCAT family members, may alter spatial regulation of KRAS and other small GTPases.

## Data availability

All data are contained within the article.

## Supplemental data

This article contains supplemental data.

## Conflict of interest

The authors declare that they have no conflicts of interest with the contents of this article.

## Supplemental data

Supplemental Figures Supplemental Figure S1. Stable expression of LPCAT1 was verified in mammalian cells. Whole-cell lysates of BHK (A), MiaPaCa-2 (B and C), MOH (C), and BxPC3 (D) cells stably expressing V2 empty vector, *LPCAT1* or sgRNA of *LPCAT1* were collected for Western blotting. Sample blots with protein ladders are shown. Supplemental Figure S2. LPCAT1 differentially modulates lipid profiles of MiaPaCa-2 cells. Whole-cell lysates of MiaPaCa-2 cells stably expressing V2 or *LPCAT1* were collected for lipidomics. A, B, C, D, E, F, G, H, I, J, and K: Levels of various lipid types with different numbers of double bonds are shown. Data are shown as mean ± SEM from 3 independent experiments. Student’s t-test was used to evaluate the statistical significance with ∗ indicating *p* < 0.05. Supplemental Figure S3. *LPCAT1* expression elevates the saturated PC and PE levels in MiaPaCa-2 cells. Individual species of the saturated PC (A) and PE (B) are shown. C: Individual PS species in MiaPaCa-2 cells expressing V2 or *LPCAT1* are shown. Data are shown as mean ± SEM from 3 independent experiments. Student’s t-test was used to evaluate the statistical significance with ∗ indicating *p* < 0.05. Supplemental Figure S4. Electron microscopy (EM)-nanoclustering analysis quantifies extent of nanoclustering of KRAS mutants. Intact apical PM sheets of BHK or MiaPaCa-2 cells stably expressing V2 or *LPCAT1* transiently expressing GFP-KRAS mutants were attached to EM grids. GFP anchored to the PM inner leaflet was immunolabeled with anti-GFP antibody conjugated to 4.5 nm gold nanoparticles. Distribution of the gold-labeled GFP-KRASG12C and GFP-KRASG12D within a selected 1μm2 PM area was calculated using the Ripley’s K-function analysis (A, B, and C). A nanoclustering curve was plotted as the extent of nanoclustering, L(r) – r, vs. length scale, r in nanometers (D). The peak value of the curve, termed as Lmax, was used as a summary statistic to indicate nanoclustering. The L(r) – r of 1 is the 99% confidence interval (99% CI, green line), the values above which indicate statistically meaningful clustering. E, F, and G: Gold nanoparticles in the same EM images shown in A, B, and C were color-coded based on their population distribution calculated in the Ripley’s K-function analysis. Supplemental Figure S5. Sample Western blotting of effects of *LPCAT1* expression on signaling of human PDAC cells. Effects of V2 vector control and LPCAT1 on MAPK (A, B, C, and D), PI3K (E, F, G, and H and loading control of β-actin (I, J, K, andL) are shown with protein ladders. Supplemental Figure S6. *LPCAT1* expression correlates with *KRAS* mutant expression in pancreatic adenocarcinoma but not lung adenocarcinoma. Statistical analyses were performed using patient data obtained from The Cancer Genomic Atlas (TCGA) in the Genomic Data Commons (GDC) data portal. *LPCAT1* mRNA levels were compared in pancreatic adenocarcinoma (A) or lung adenocarcinoma (B) patients with the KRAS-dependent and KRAS-independent tumors. Welch’s t-tests were performed to evaluate the statistical significance. *Lpcat1* expression was then correlated with mutant *KRAS* expression in PAAD (C) and LUAD (D). Pearson's rho and Spearman's rank rho were calculated. Statistical analyses were conducted using the University of ALabama at Birmingham CANcer data analysis Portal (UALCAN). LPCAT1 protein expression levels were compared between normal and tumor tissues in pancreatic adenocarcinoma (E) and lung adenocarcinoma (F).
