# Supplementary material for: KRAS G12C and KRAS G12D respond to lipid metabolism in an allele-specific manner
Source: J Lipid Res. 2026 Jun 16;67(7):101079. doi: 10.1016/j.jlr.2026.101079 (PMC13375922; doi:10.1016/j.jlr.2026.101079)

Supplemental Figure 1

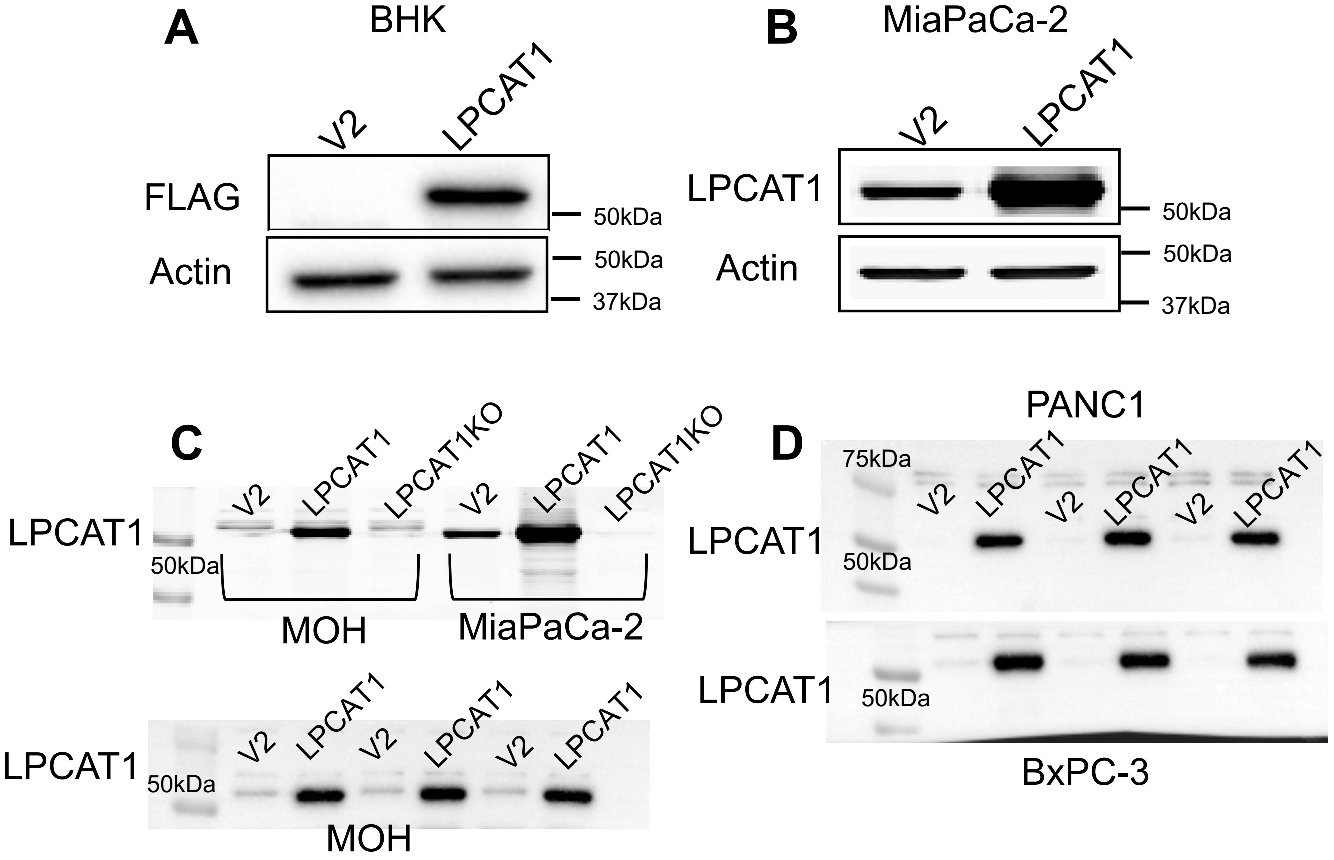

Supplemental Figure 2

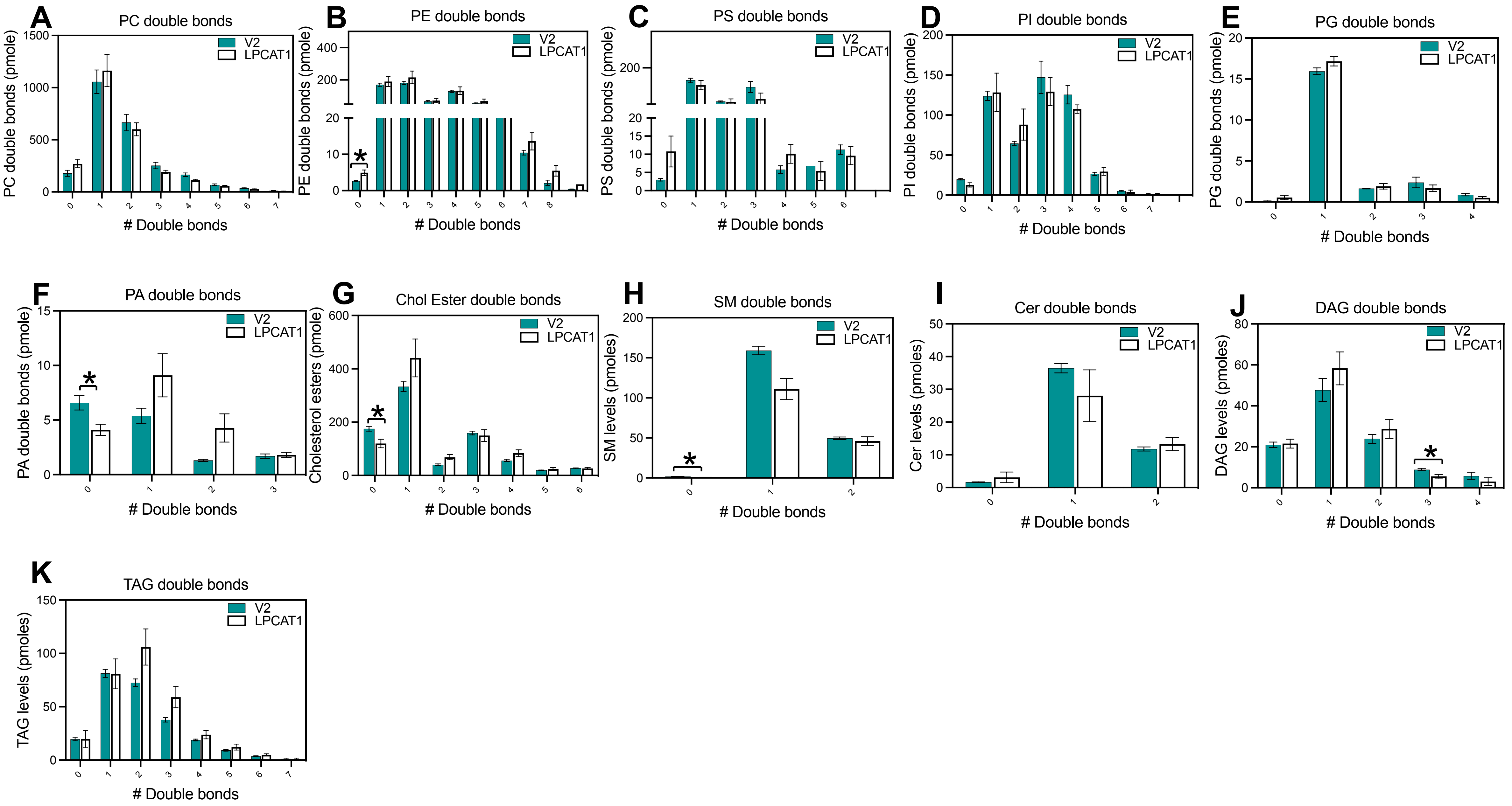

Supplemental Figure 3

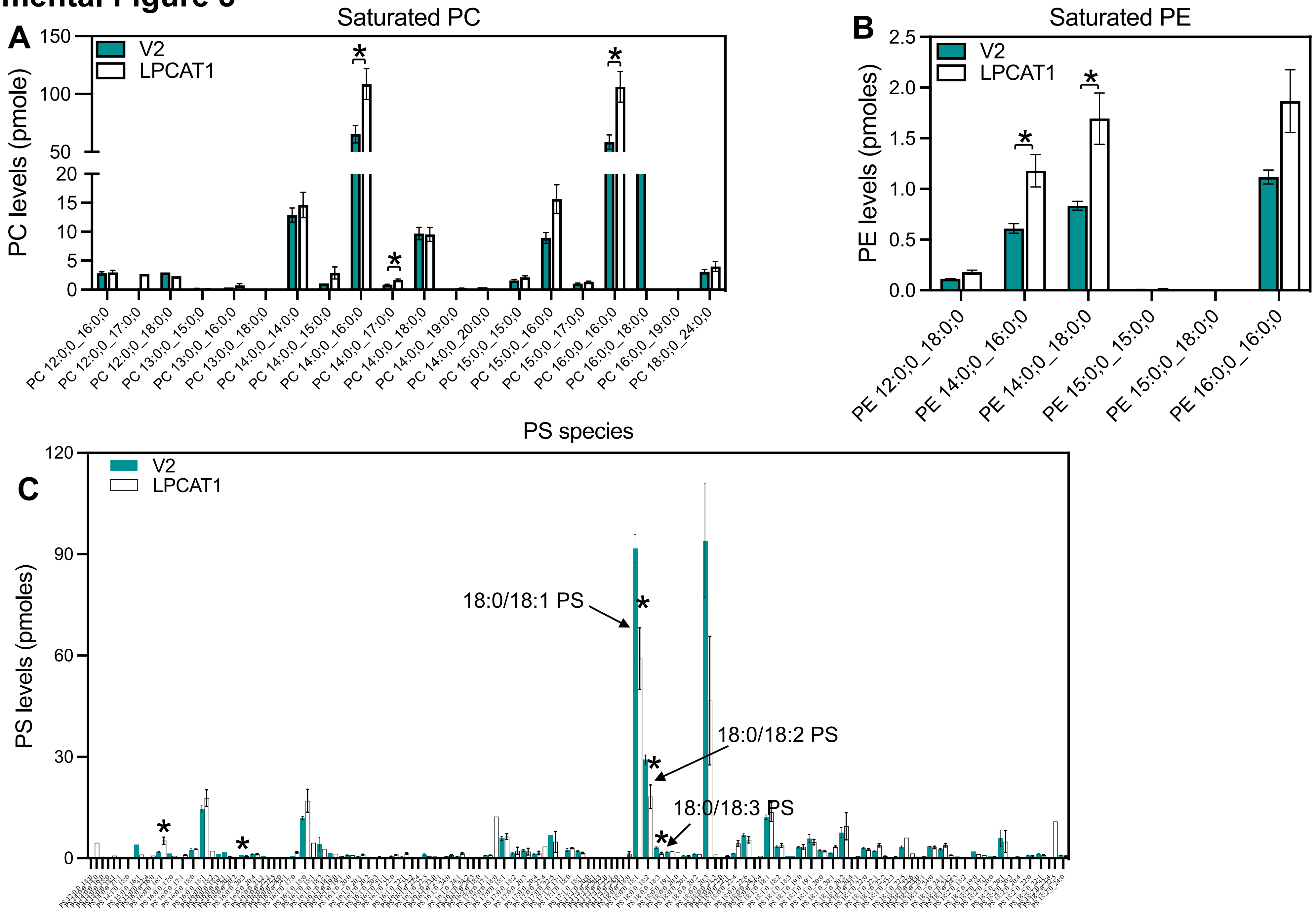

Supplemental Figure 4

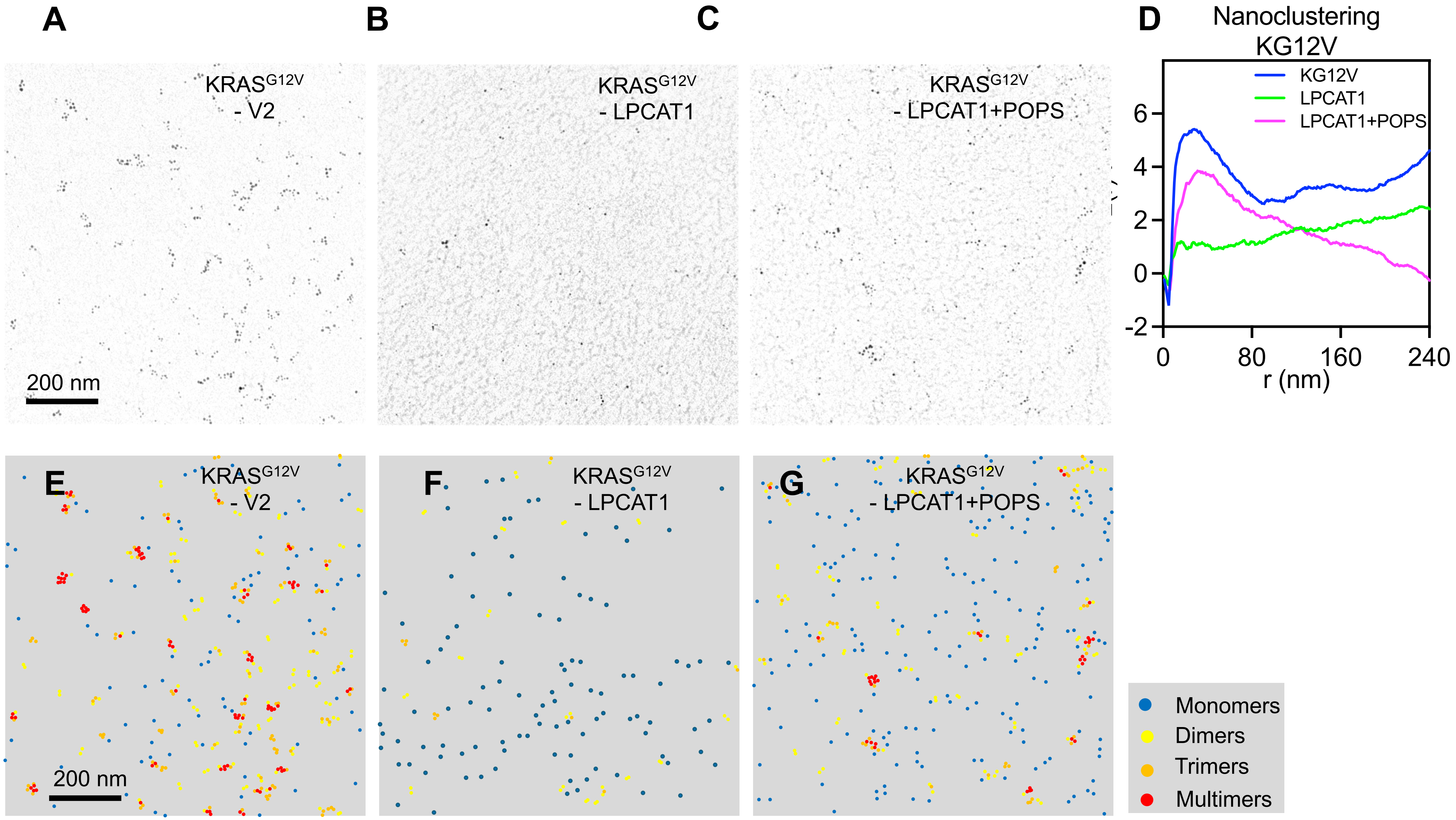

Supplemental Figure 5

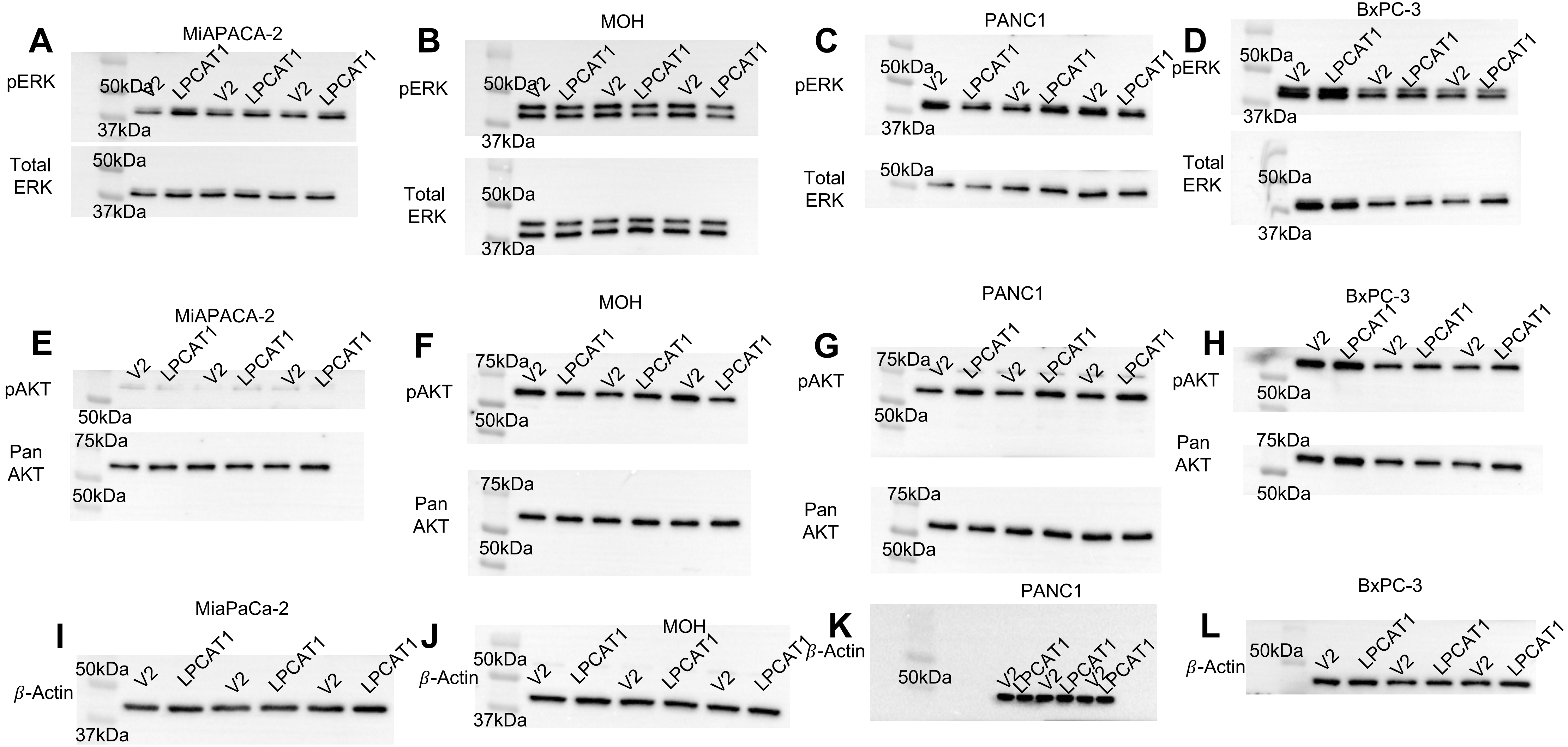

Supplemental Figure 6

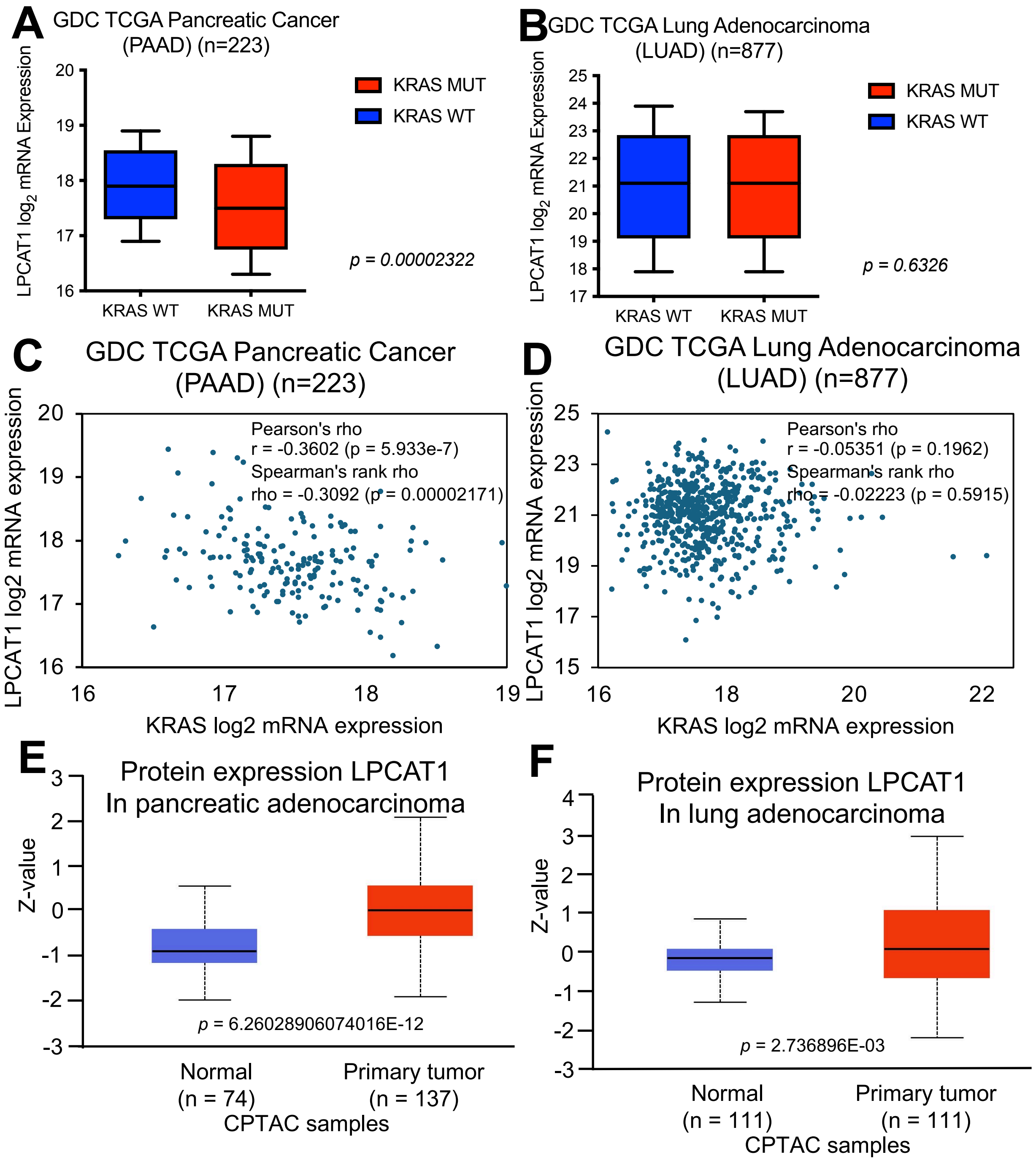

Supplement: Supplemental Figures — Supplemental Figure S1. Stable expression of LPCAT1 was verified in mammalian cells. Whole-cell lysates of BHK (A), MiaPaCa-2 (B and C), MOH (C), and BxPC3 (D) cells stably expressing V2 empty vector, LPCAT1 or sgRNA of LPCAT1 were collected for Western blotting. Sample blots with protein ladders are shown. Supplemental Figure S2. LPCAT1 differentially modulates lipid profiles of MiaPaCa-2 cells. Whole-cell lysates of MiaPaCa-2 cells stably expressing V2 or LPCAT1 were collected for lipidomics. A, B, C, D, E, F, G, H, I, J, and K: Levels of various lipid types with different numbers of double bonds are shown. Data are shown as mean ± SEM from 3 independent experiments. Student’s t-test was used to evaluate the statistical significance with ∗ indicating p < 0.05. Supplemental Figure S3. LPCAT1 expression elevates the saturated PC and PE levels in MiaPaCa-2 cells. Individual species of the saturated PC (A) and PE (B) are shown. C: Individual PS species in MiaPaCa-2 cells expressing V2 or LPCAT1 are shown. Data are shown as mean ± SEM from 3 independent experiments. Student’s t-test was used to evaluate the statistical significance with ∗ indicating p < 0.05. Supplemental Figure S4. Electron microscopy (EM)-nanoclustering analysis quantifies extent of nanoclustering of KRAS mutants. Intact apical PM sheets of BHK or MiaPaCa-2 cells stably expressing V2 or LPCAT1 transiently expressing GFP-KRAS mutants were attached to EM grids. GFP anchored to the PM inner leaflet was immunolabeled with anti-GFP antibody conjugated to 4.5 nm gold nanoparticles. Distribution of the gold-labeled GFP-KRASG12C and GFP-KRASG12D within a selected 1μm2 PM area was calculated using the Ripley’s K-function analysis (A, B, and C). A nanoclustering curve was plotted as the extent of nanoclustering, L(r) – r, vs. length scale, r in nanometers (D). The peak value of the curve, termed as Lmax, was used as a summary statistic to indicate nanoclustering. The L(r) – r of 1 is the 99% confidence interva [file mmc1.pdf]
